# Molecular characteristics and genetic evolutionary analyses of circulating parvoviruses derived from cats in Beijing

**DOI:** 10.1186/s12917-022-03281-w

**Published:** 2022-05-23

**Authors:** Yashu Tang, Na Tang, Jingru Zhu, Min Wang, Yang Liu, Yanli Lyu

**Affiliations:** 1 Key Laboratory of Animal Epidemiology of the Ministry of Agriculture, College of Veterinary Medicine, China Agricultural University, Beijing, 100193 China; 2 Department of Clinical Veterinary Medicine, College of Veterinary Medicine, China Agricultural University, Beijing, 100193 China; 3 College of Veterinary Medicine, Veterinary Teaching Hospital, China Agricultural University, Beijing, 100193 China

**Keywords:** Feline parvovirus (FPV), Canine parvovirus (CPV), VP2 gene, Evolution, Recombination

## Abstract

**Background:**

Feline parvovirus (FPV) is a member of the family *Parvoviridae*, which is a major enteric pathogen of cats worldwide. This study aimed to investigate the prevalence of feline parvovirus in Beijing of China and analyze the genetic features of detected viruses.

**Results:**

In this study, a total of 60 (8.5%) parvovirus-positive samples were detected from 702 cat fecal samples using parvovirus-specific PCR. The complete VP2 genes were amplified from all these samples. Among them, 55 (91.7%) sequences were characterized as FPV, and the other five (8.3%) were typed as canine parvovirus type 2 (CPV-2) variants, comprised of four CPV-2c and a new CPV-2b strain. In order to investigate the origin of CPV-2 variants in cats, we amplified full-length VP2 genes from seven fecal samples of dogs infected with CPV-2, which were further classified as CPV-2c. The sequences of new CPV-2b/MT270586 and CPV-2c/MT270587 detected from feline samples shared 100% identity with previous canine isolates KT156833 and MF467242 respectively, suggesting the CPV-2 variants circulating in cats might be derived from dogs. Sequence analysis indicated new mutations, Ala91Ser and Ser192Phe, in the FPV sequences, while obtained CPV-2c carried mutations reported in Asian CPV variants, showing they share a common evolutionary pattern with the Asian 2c strains. Interestingly, the FPV sequence (MT270571), displaying four CPV-specific residues, was found to be a putative recombinant sequence between CPV-2c and FPV. Phylogenetic analysis of the VP2 gene showed that amino acid and nucleotide mutations promoted the evolution of FPV and CPV lineages.

**Conclusions:**

Our findings will be helpful to further understand the circulation and evolution of feline and canine parvovirus in Beijing.

## Background

Feline parvovirus infection is a contagious disease characterized by severe leukopenia, vomiting, diarrhea with fever and abdominal pain, and a high rate of morbidity in young cats [1]. This disease is caused by feline parvovirus (FPV), a small, non‐enveloped single‐stranded DNA virus. FPV is a member of the family *Parvoviridae*, subfamily *Parvovirinae*, genus *Protoparvovirus*, and it was recently included in the unique species *Carnivore protoparvovirus 1*, together with canine parvovirus (CPV), mink enteritis virus (MEV), and raccoon parvovirus (RPV) [2].

Recent studies showed that in domestic felines, FPV remains the prevalent cause of parvovirus infection, while CPV infection became increasingly common. Molecular surveillance in China demonstrated that FPV and CPV variants (CPV-2a/2b and new CPV-2a/2b) are co-circulating in cats in Northeast China [3], while new CPV-2a was the predominant CPV variant in domestic cats in Beijing [4] and Henan province [5]. After its emergence, CPV-2c was found with high incidence in the European feline population, but was relatively rare in Asia [6–9]. In China, CPV-2c infection was first reported in Jilin province in 2009 [10]. Later, this variant was also identified in dogs from other provinces [11–13]. However, there has not been any report of CPV-2c infection from cats in China before.

Although showed a completely different pattern, genetic recombination played an important role in the evolution of both FPV and CPV [14–16]. In FPV, evolution was mainly forced by random genetic drift [17]. The antigenic and biological properties of FPV have not experienced significant changes since its first identification in 1920 [18]. On the contrary, CPV evolved by positive selection and high nucleotide substitution rate [19], which leads to the emergence of new antigenic variants (2a, 2b, and 2c) and enables them to expand their host range to cats. Genetic recombination is generally considered as a major key mechanism for virus evolution, especially for RNA viruses such as feline calicivirus [20], feline immunodeficiency virus [21], and canine distemper virus [22]. Recently, Shackelton et al*.* pointed out that genetic recombination should be considered as an essential mechanism for the evolution of parvoviruses in nature [19]. Subsequently, they described natural recombination among porcine parvovirus, Aleutian mink disease virus, and several rodent parvoviruses [23]. More recently, studies in Japan, Uruguay, and China reported the natural recombination between the vaccine CPV-2 and either field CPV-2a or 2b [14], CPV-2c and CPV-2a [16] as well as FPV and CPV-2 [15], respectively, further indicating the important role of genetic recombination in the natural evolution of parvoviruses. Moreover, some recombination events possibly went undetected due to the high genome similarity (98%) between FPV and CPV.

Previous studies provided information on FPV and CPV strains spreading in domestic cats in some provinces of China, while there was limited information regarding parvovirus in cats, suggesting the need for an epidemiological survey to evaluate the parvovirus circulation and evolution in the cat population in China. In this study, we characterized the nucleotide sequences and key amino acid sites of FPV and CPV VP2 gene collected from domestic cats in Beijing. We also compared the VP2 sequences of CPV from cats to those from dogs in Beijing area. This helps us to investigate the prevalence of parvovirus among cats in Beijing and to gain insights into the evolution of the detected FPV and CPV viruses.

## Results

### Detection and characterization of VP2 gene

Sixty (8.5%) samples from 702 cats and seven samples from dogs were tested positive for parvovirus. The complete VP2 sequences were amplified from the parvovirus positive samples, and all of them are 1755 bp in length. They were submitted to GenBank database under accession numbers MT270531-MT270590 (feline sequences) and MT270591-MT270597 (canine sequences). Analysis of the key aa residues of the VP2 protein showed that the 60 sequences from cats were typed as FPV (91.7%; *n* = 55), new CPV-2b (1.7%; *n* = 1) or CPV-2c (6.7%; *n* = 4). No type CPV-2, CPV-2a/2b or new CPV-2a was found in this study. Meanwhile, seven sequences from dogs were all carried residue 426Glu and therefore were typed as CPV-2c variants (Table 1). In addition, almost all the sequences obtained from cats with gastrointestinal symptoms, the two exceptions being an FPV sequence (MT270580) detected from a cat suffering from bone fracture and a CPV-2c sequence (MT279589) detected from a healthy cat.

**Table 1:** Mutations of nucleotides and amino acids in the VP2 sequences of the viral strains

Accession number	Origin	VP2 nucleotide/amino acid position^a^													Type
		**14**	**271**	**575**	**694&696**	**800**	**889**	**899**	**970&971**	**1109**	**1276&1278**	**1318**	**1691**	**1703**	
		**5**	**91**	**192**	**232**	**267**	**297**	**300**	**324**	**370**	**426**	**440**	**564**	**568**	
**M24004**	**reference**	C/Ala	G/Ala	C/Ser	A&A/Ile	T/Phe	T/Ser	C/Ala	T&A/Tyr	A/Gln	A&T/Asn	A/Thr	A/Asn	C/Ala	**FPV**
**M38245**	**reference**	C/Ala	G/Ala	C/Ser	A&A/Ile	T/Phe	T/Ser	C/Ala	T&A/Tyr	A/Gln	A&T/Asn	A/Thr	G/Ser	G/Gly	**CPV-2**
**M74849**	**reference**	C/Ala	G/Ala	C/Ser	A&A/Ile	T/Phe	T/Ser	G/Gly	T&A/Tyr	A/Gln	G&T/Asp	A/Thr	G/Ser	G/Gly	**CPV-2b**
**FJ222821**	**reference**	C/Ala	G/Ala	C/Ser	A&A/Ile	T/Phe	G/Ala	G/Gly	T&A/Tyr	A/Gln	G&A/Glu	A/Thr	G/Ser	G/Gly	**CPV-2c**
**MT270585**	**cat**	**-**	**-**	**-**	G&A/Val	**-**	**-**	**-**	**-**	**-**	**-**	**-**	**-**	**-**	**FPV**
**MT270584**	**cat**	**-**	**-**	**-**	G&A/Val	**-**	**-**	**-**	**-**	**-**	**-**	**-**	**-**	**-**	**FPV**
**MT270583**	**cat**	**-**	**-**	**-**	G&A/Val	**-**	**-**	**-**	**-**	**-**	**-**	**-**	**-**	**-**	**FPV**
**MT270582**	**cat**	**-**	**-**	**-**	G&A/Val	**-**	**-**	**-**	**-**	**-**	**-**	**-**	**-**	**-**	**FPV**
**MT270581**	**cat**	**-**	T/Ser	**-**	G&A/Val	**-**	**-**	**-**	**-**	**-**	**-**	**-**	**-**	**-**	**FPV**
**MT270578**	**cat**	**-**	**-**	**-**	G&A/Val	**-**	**-**	**-**	**-**	**-**	**-**	**-**	**-**	**-**	**FPV**
**MT270577**	**cat**	**-**	**-**	**-**	G&A/Val	**-**	**-**	**-**	**-**	**-**	**-**	**-**	**-**	**-**	**FPV**
**MT270576**	**cat**	**-**	T/Ser	T/Phe	G&A/Val	**-**	**-**	**-**	**-**	**-**	**-**	**-**	**-**	**-**	**FPV**
**MT270575**	**cat**	**-**	T/Ser	**-**	G&A/Val	**-**	**-**	**-**	**-**	**-**	**-**	**-**	**-**	**-**	**FPV**
**MT270574**	**cat**	**-**	T/Ser	**-**	G&A/Val	**-**	**-**	**-**	**-**	**-**	**-**	**-**	**-**	**-**	**FPV**
**MT270571**	**cat**	**-**	T/Ser	**-**	G&A/Val	**-**	**-**	G/Gly	**-**	**-**	G&A/Glu	**-**	G/Ser	G/Gly	**FPV**
**MT270570**	**cat**	**-**	**-**	**-**	G&A/Val	**-**	**-**	**-**	**-**	**-**	**-**	**-**	**-**	**-**	**FPV**
**MT270569**	**cat**	**-**	T/Ser	**-**	G&A/Val	**-**	**-**	**-**	**-**	**-**	**-**	**-**	**-**	**-**	**FPV**
**MT270566**	**cat**	**-**	**-**	**-**	G&A/Val	**-**	**-**	**-**	**-**	**-**	**-**	**-**	**-**	**-**	**FPV**
**MT270559**	**cat**	**-**	T/Ser	**-**	G&A/Val	**-**	**-**	**-**	**-**	**-**	**-**	**-**	**-**	**-**	**FPV**
**MT270544**	**cat**	**-**	T/Ser	**-**	G&A/Val	**-**	**-**	**-**	**-**	**-**	**-**	**-**	**-**	**-**	**FPV**
**MT270543**	**cat**	**-**	**-**	**-**	G&A/Val	**-**	**-**	**-**	**-**	**-**	**-**	**-**	**-**	**-**	**FPV**
**MT270540**	**cat**	**-**	T/Ser	**-**	G&A/Val	**-**	**-**	**-**	**-**	**-**	**-**	**-**	**-**	**-**	**FPV**
**MT270538**	**cat**	**-**	T/Ser	**-**	G&C/Val	**-**	**-**	**-**	**-**	**-**	**-**	**-**	**-**	**-**	**FPV**
**MT270534**	**cat**	**-**	**-**	**-**	G&A/Val	**-**	**-**	**-**	**-**	**-**	**-**	**-**	**-**	**-**	**FPV**
**MT270586**	**cat**	**-**	**-**	**-**	**-**	A/Tyr	G/Ala	-	A&T/Ile	-	-	G/Ala	-	**-**	**New CPV-2b**
**MT270590**	**cat**	G/Gly	**-**	**-**	**-**	A/Tyr	**-**	**-**	A&T/Ile	G/Arg	**-**	**-**	**-**	**-**	**CPV-2c**
**MT270589**	**cat**	G/Gly	**-**	**-**	**-**	A/Tyr	**-**	**-**	A&T/Ile	G/Arg	**-**	**-**	**-**	**-**	**CPV-2c**
**MT270588**	**cat**	G/Gly	**-**	**-**	**-**	A/Tyr	**-**	**-**	A&T/Ile	G/Arg	**-**	**-**	**-**	**-**	**CPV-2c**
**MT270587**	**cat**	G/Gly	**-**	**-**	**-**	A/Tyr	**-**	**-**	A&T/Ile	G/Arg	**-**	**-**	**-**	**-**	**CPV-2c**
**MT270597**	**dog**	G/Gly	**-**	**-**	**-**	A/Tyr	**-**	**-**	A&T/Ile	G/Arg	**-**	**-**	**-**	**-**	**CPV-2c**
**MT270596**	**dog**	G/Gly	**-**	**-**	**-**	A/Tyr	**-**	**-**	A&T/Ile	G/Arg	**-**	**-**	**-**	**-**	**CPV-2c**
**MT270593**	**dog**	G/Gly	**-**	**-**	**-**	A/Tyr	**-**	**-**	A&T/Ile	A/Gln	**-**	**-**	**-**	**-**	**CPV-2c**
**MT270592**	**dog**	G/Gly	**-**	**-**	**-**	A/Tyr	**-**	**-**	A&T/Ile	G/Arg	**-**	**-**	**-**	**-**	**CPV-2c**
**MT270591**	**dog**	G/Gly	**-**	**-**	**-**	A/Tyr	**-**	**-**	A&T/Ile	G/Arg	**-**	**-**	**-**	**-**	**CPV-2c**

*Note*: In order to simplify the presentation of results, the identical sequences at the nucleotide level have not been included in the table. Sites where no variation was observed are marked by "-"

^a^Nucleotide and amino-acid positions refer to the prototype FPV (M24004) and CPV-2 (M38245)

Nucleotide pairwise identity revealed that the 55 FPV VP2 sequences in this study were highly identical, ranging from 98.6% to 100.0% in nucleotide similarity, and sixteen of them were 100% identical. However, the VP2 gene from cats here showed genetic diversity between FPV and CPV detected in this study, with 97.7%-98.6% nucleotide identities. Interestingly, the VP2 sequence of CPV-2c from cats were closely related to those from dogs in this study, sharing a nucleotide identity of 99.7–100%. Moreover, the two CPV-2c VP2 genes, MT270587 and MT270589, were completely identical to MT270594 and MT270595 from dogs, respectively. Furthermore, the CPV-2c VP2 gene (MT270587) from a cat also shared 100% identity with that of a CPV-2c strain (MF467242) isolated from a dog in Guangxi of China in 2015. Meanwhile, the new CPV-2b VP2 gene (MT270586) from a cat in this study showed 100% identity with the strain (KT156833) derived from a dog in Heilongjiang of China in 2014.

### Sequence analysis of VP2 gene

Analysis of deduced amino acid (aa) sequences of the FPV VP2 gene revealed that all the 55 (100%) FPV sequences had the Ile232Val mutation, 39 (70.9%) sequences had the Ala91Ser mutation, and one (1.8%) had the Ser192Phe mutation (Table 1). Notably, FPV/MT270571 (BJ-A240) was detected as a highly different sequence, with one 2c-specific residue, Asn426Glu, and three 2a/2b/2c-specific residues Ala300Gly, Asn564Ser and Ala568Gly (Table 1).

When compared with the reference strains, six aa mutations were observed in the CPV VP2 genes (Table 1). The derived VP2 amino acid sequences of the 11 CPV-2c and the new CPV-2b presented Phe267Tyr and Tyr324Ile mutations, which were observed in 100% of recently analyzed Asian CPVs [13]. Mutations Ser297Ala and Thr440Ala detected in the new CPV-2b sequence from this study, as well as the Ala5Gly and Gln370Arg in the 11 CPV-2c sequences (from cats and dogs), were identical to recent Asian new CPV-2b [13] and CPV-2c strains [13, 24, 25], respectively.

### Phylogeny

**Fig. 1:**
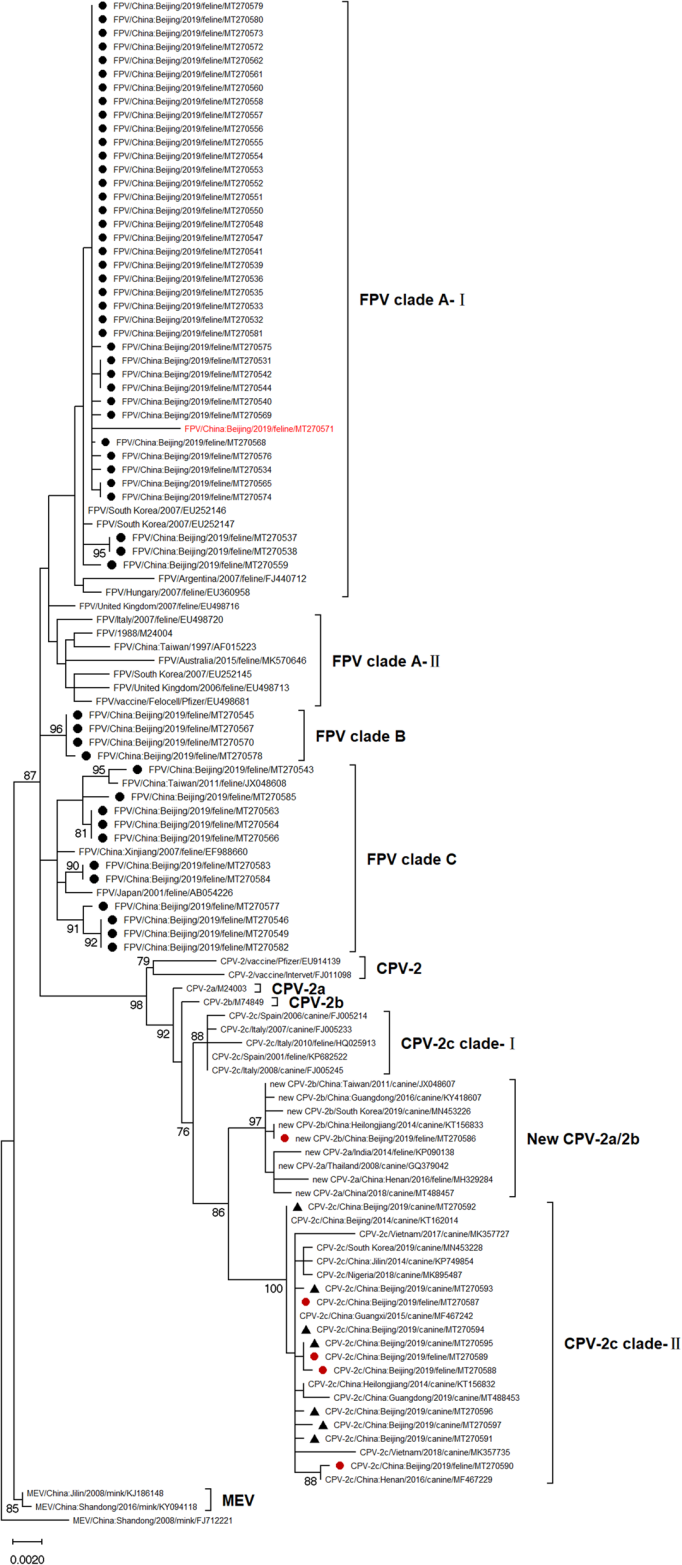
Maximum-likelihood tree showing the genetic relationship of the full-length VP2 gene of feline parvovirus and canine parvovirus strains. Maximum‐likelihood (ML) tree based on 112 full‐length VP2 sequences of FPV, CPV and MEV strains. The tree was constructed using the T92 + G model and 1,000 bootstrapping with MEGAX software. Mink enteritis virus (MEV) was used as the outgroup. Bootstrap values (%) greater than 50 are shown. Sequences used in this analysis are indicated with their respective virus type (FPV/CPV/MEV) or variant (CPV-2/2a/2b/2c, new CPV-2a/2b), country and year of collection, origin, and GenBank accession number. FPV sequences detected in this study are indicated by black dots and CPV sequences from cats and dogs are indicated by red dots and black triangles, respectively. The recombinant FPV sequence MT270571 was shown in red

On phylogenetic analysis, sixty-seven VP2 nucleotide sequences from this study and fifty-four from GenBank clustered mainly depending on their virus type, formed three different branches, FPV, CPV, and MEV branch (Fig. 1). In the FPV branch, the 70 VP2 sequences fell into three major clades, clade A, B, and C. The clade A consisted of 52 sequences with 1185 T and clade B included four sequences with A at nucleotide residue 1185, while sequences presented 1185C clustered separately in clade C. Thereafter, the FPV clade A was further divided into two small clades according to several synonymous substitutions in VP2 gene. Forty obtained FPV sequences, presented 271 T and 1041A, segregated in the A-I clade. Also, seven FPV sequences grouped in the clade A-II, and they all had 271G and 1041G. Furthermore, the clade A-I also subdivide into Asian subclade and European subclade (FJ440712 and EU360958) depending on the nucleotide residues 750 and 1572.

In addition, the 39 CPV sequences clustered into six clades according to the variants type of CPV (Fig. 1). Twenty-one CPV-2c sequences expressed 267Tyr, 324Ile, and 370Arg in VP2, forming the 2c Asian subclade (2c clade-II), which included 2c-type sequences obtained from cats and dogs in this study. Five strains showed 267Phe, 324Tyr, and 370Gln separated in the 2c European subclade (2c clade-I), contained CPV-2c detected from Spain and Italy. However, the MT270593 displayed 370Gln in VP2 protein, clustered in the 2c Asian clade. During the last few decades, CPV-2a/2b carrying Ser297Ala were designated new CPV-2a/2b [26, 27]. Therefore, eight of the variants that had been initially named as CPV‐2a/2b in the NCBI database were updated to new CPV‐2a/2b in the ML phylogenetic tree. Consequently, the new CPV-2b sequences (from China and South Korea) and new CPV-2a isolates (from China, Thailand and India) established the new CPV-2a/2b clade based on the Ser297Ala change, distinguished from the reference VP2 sequences of CPV-2a (M24003) and CPV-2b (M74849).

### Recombination analyses

**Fig. 2:**
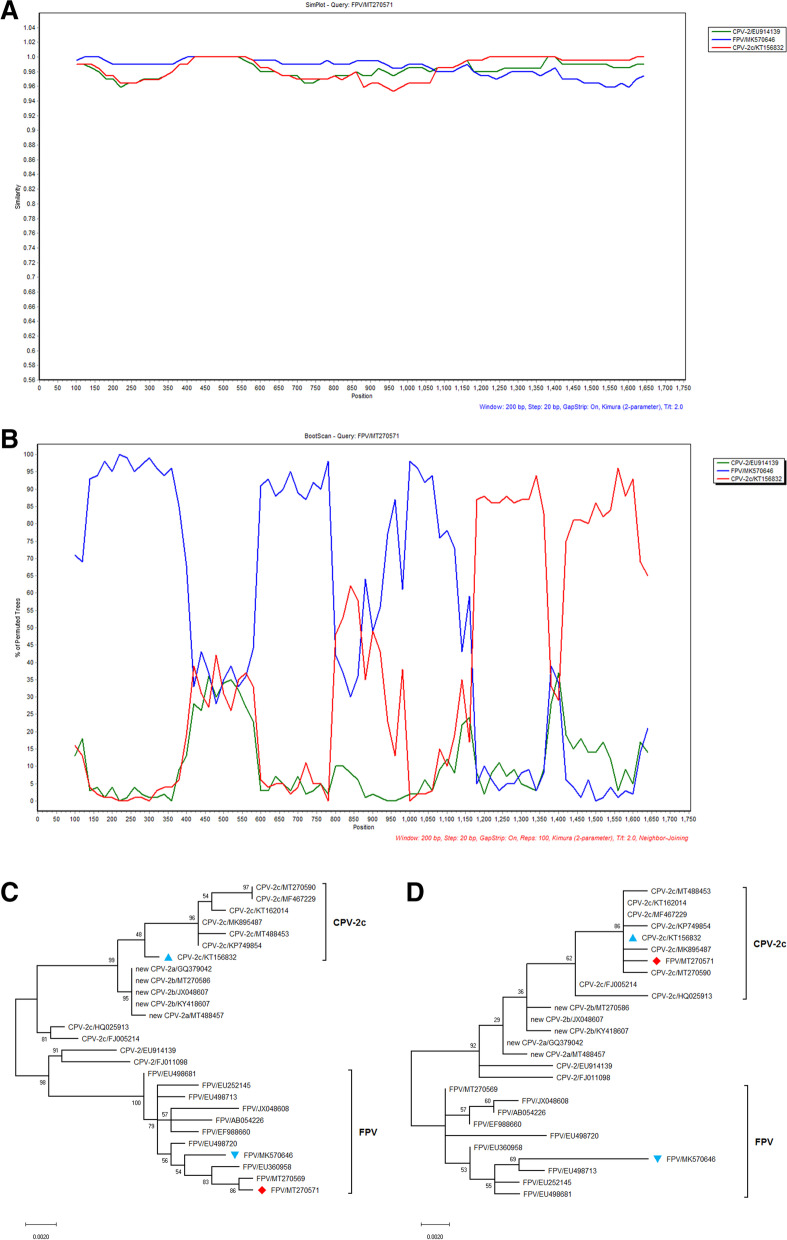
Schematic diagram of the naturally recombinant FPV/MT270571 sequence. CPV-2c/KT156832 isolated in China and FPV/MK570646 from Australia served as the putative major and minor parents. **A** The potential recombination event was detected in the VP2 protein gene and was supported by similarity (2A) and bootscan (2B) analysis, which indicated that CPV-2c/KT156832 (red line) served as the main template of the complete VP2 gene, and the beginning of the VP2 gene was replaced by FPV/MK570646 (blue line). The FPV/MT270571 sequence served as the query. The y-axis indicated the percentage of nucleotide identity and permutated trees for the similarity plot and boot scanning, respectively, within a 200 bp-wide window with a 20-bp step size between plots. **B** The ML phylogenetic trees of the recombinant MT/270571 strains (♦) and it's major (▲) and minor (▼) putative parent strains over nucleotides 1–1,129 (2C) and 1,130–1,755 (2D). Bootstrap (1000 replications) values over 50% are shown for each node

With respect to the genetic recombination forced evolution of parvovirus [19], two genetic recombination detection modules annotated the FPV/MT270571 as a potential recombinant sequence. The RDP4 identified evidence of genetic recombination in the VP2 protein of FPV/MT270571, supported by Maxchi, SiScan, and 3Seq with a *p*-value of 4.263 × 10^−3^, 4.085 × 10^−6^, and 8.115 × 10^−4^, respectively. The recombinant FPV/MT270571 sequence had a CPV-2c strain KT156832 isolated from a dog in China and a FPV strain MK570646 derived from a cat in Australia as its putative major and minor parents, respectively. The obtained RDP4 results were then further examined using the SimPlot software, which confirmed that the FPV/MT270571 was a recombinant sequence. The generated similarity plot and bootscan analysis for the FPV/MT270571 sequence suggested it had a high nucleotide similarity to the FPV strain MK570646 (blue line) at the beginning of the VP2 gene, but also had high nucleotide identity to the CPV-2c strain KT156832 (red line) at the latter part of VP2 gene (Fig. 2A&2B). The VP2 sequences were divided into two alignments around the site of the breakpoint (nt 1129), and separate phylogenetic trees for each dataset were constructed, which further confirmed the potential recombination event in FPV/MT270571 (Fig. 2C&D).

## Discussion

In recent years, the total number of cats infected with FPV has increased with the expansion of pet cat population despite the widespread use of vaccines in Beijing. This study revealed that cats in Beijing are still primarily infected by FPV strains, along with the new CPV-2b and CPV-2c variants co-circulating in the population. It also showed that CPV-2c strain has emerged for the first time as a dominant antigenic CPV variant prevalent in cats in Beijing. Recently, studies have shown that the new CPV-2a variants were prevalent in dogs in some provinces of China, including Beijing (2014–2015) [11], Sichuan (2011) [28] Gansu (2014) [29], Shandong (2015) and Heilongjiang (2014–2015) [13], whereas CPV‐2c infection hasn't been reported yet. However, this situation has changed as the detection of seven CPV-2c sequences from seven dogs in this study, suggesting the 2c variant probably has replaced new CPV-2a become the predominant strain in Beijing.

Moreover, we found that the CPV-2c sequence (MT270587) obtained from a cat displayed 100% identity with the CPV-2c sequence (MT270594) detected from a dog in this study as well as a published 2c-type sequence (MF467242) from a dog in Guangxi of China in 2015, which is consistent with previous studies by Decaro et al*.* [6] and Wu et al*.* [4]. Meanwhile, the CPV-2c sequence (MT270589) and the new CPV-2b sequence (MT270586), detected from cats, were identical to the obtained sequence (CPV-2c/MT270595) and a reported sequence (new CPV-2b/KT156833) from dogs in Heilongjiang of China, respectively. These results indicated that the 2c-type sequences are circulating in dog population in China, and the CPV sequences in cats were derived from dogs. In China, many families own both dogs and cats and they often share a room during long-distance transportation. Therefore, the risk of canine viruses spread through different areas and species has been increased, which may cause the infections of previous canine isolates in cats discovered in this study.

Based on the sequence analysis, Ala91Ser, and Ser192Phe were first identified in FPV VP2 sequences. Besides, all FPV sequences tested in this study carried a reported [30] mutation, Ile232Val, which probably represented a novel pattern of VP2 genetic evolution in FPV strains in Beijing. The potential functional consequence of these mutations remains unknown. But several synonymous substitutions worked as the hallmark to separate FPV sequences in the phylogenetic analysis, as shown in Fig. 1. On the other hand, mutations observed in obtained CPV-2c and new CPV-2b VP2 sequences have been elaborated in other CPV variants [11–13, 24, 26–29, 31–43]. Among these, Phe267Tyr, Tyr324Ile, Gln370Arg mutations served as the evolutionary force, further divided the 2c clade into Asian/European subclades. Moreover, the exceptions in these geographically distinct clusters may also result from animal trading such as transporting infected animals between different areas or transporting healthy animals in contaminated equipment [44]. Taken together, changes in the VP2 gene might play a significant role in the evolution of FPV and CPV viruses.

Interestingly, we detected high genetic complexity of the sequence FPV/MT270571, which carried four CPV-specific residues. This sequence was further subjected to recombination analysis, which was proven to be a recombinant sequence between CPV-2c and FPV in VP2 gene. Recently, several studies have reported the natural recombination events between the vaccine CPV-2 and either field CPV-2a or 2b [14], FPV and new CPV-2b [15], as well as CPV-2c and CPV-2a [16], showing the important role of genetic recombination in the natural evolution of parvoviruses. In the present study, the MT270571 sequence was most closely related to FPV/MK570646, indicating the likely origin (strain) from which the 1–1,129 nt region in the VP2 gene of the recombinant MT270571 circulating in Beijing came from. Furthermore, as cats are susceptible to both FPV and CPV variants [45, 46], co-infection with multiple parvovirus strains occurred [1, 9, 47], potentially facilitating recombination and high genetic heterogeneity. Therefore, we assumed that recombination during co-infection of FPV and CPV-2c viruses in a cat was the most likely origin of this recombinant FPV VP2 gene, and its putative minor parent sequence (FPV/MK570646) may be introduced from Australia through the imported dog.

To our knowledge, this is the first demonstration of FPV and CPV-2c recombination within VP2 genes in field. However, the effects of this mutation on FPV strains are unknown. Previously identified recombination breakpoints occur within the VP1/VP2 [16] or NS1/VP1 [15] gene boundary, therefore ongoing researches on the potential recombinant events in the NS and VP1 genes of sample BJ-A240 are necessary.

## Conclusion

In conclusion, these results revealed that FPV is still the predominant parvovirus strain circulating in cat populations, and a small number of new CPV-2b, CPV-2c, and recombinant FPV strains are prevalent in Beijing. Besides, this study provided the first evidence of CPV-2c emerged as the dominant antigenic CPV variant circulating in domestic cats and dogs in Beijing, which had a common evolutionary pattern in VP2 protein with other Asian CPV-2c strains. Moreover, the FPV and CPV lineages likely evolve by changes in nt and aa composition of VP2 gene, and identification of the genetic recombination in VP2 protein may contribute to the evolution of parvovirus diversity. Further in-depth studies of the pathology of the recombinant infection are required.

## Methods

### Sample collection

A total of 702 fecal samples from both healthy and parvoviruses-suspected cats were collected at China Agricultural University Veterinary Teaching Hospital in Beijing during 2019. The observed clinical signs were depression, fever, dehydration, vomiting, and diarrhea. Meanwhile, seven samples from domestic dogs diagnosed with CPV infection using colloidal gold test strips were obtained in the same region for comparison. The fecal samples were homogenized in 1 mL of 0.1 M PBS of pH 7.4, centrifuged at 8000 g for 10 min at 4 °C, and the supernatant was collected and kept at -80 °C for later investigation.

### DNA extraction and VP2 sequencing

For each sample, DNA was extracted from specimens using the Aidlab Virus DNA Kit (Beijing Aidlab Biotech Company, Beijing, China) according to the manufacturer’s instructions. Presence of FPV/CPV in extracted viral DNA was screened by PCR using F1 primer pairs amplifying a 1325-bp fragment of the VP2 gene (Table 2). Amplification was carried out in 25-μL reactions, consisting of 12.5 μL extensor PCR master mix (Aidlab), 9.5 μL of nuclease-free water, 0.5 μL of each primer (10 μM; F1F and F1R) and 2 μL of DNA template. Negative controls (water) were processed alongside fecal samples throughout all stages. The PCR cycling conditions were 5 min at 94 °C, followed by 30 cycles of denaturation at 94ºC for 10 s, annealing at 53ºC for 15 s and extension at 72ºC for 20 s, with a final extension at 72ºC for 10 min. A 5 μL aliquot of each PCR product was analyzed by electrophoresis using a 1% agarose gel and ethidium bromide staining. Subsequently, the parvovirus PCR positive samples were further subjected to full-length VP2 sequencing using F2 primer pairs, which amplify an 817-bp fragment of the VP2 gene (Table 2). The PCR amplification and thermal cycling conditions for the F2 primers were prepared as for the F1 primers, with minor modifications: 6.5 μL of nuclease-free water, plus 3 μL of MgCl_2_ (25 mM).

**Table 2:** PCR primers used for amplification of the full-length VP2 gene of FPV and CPV

Primer	Sequence 5’ to 3’	Position^a^	Length (bp)^a^
F1F	CCACCTCATATTTTCATCAA	2709–2728	1325
F1R	TGAATCCAATCTCCTTCTG	4015–4033	
F2F	GATGAAAATCAAGCAGCAG	3885–3903	817
F2R	CCTTCTAAATCCTATATCAAATAC	4678–4701	

^a^The size and nucleotide position of the PCR products, according to the genomic sequence of CPV-2 (GenBank accession no. M38245)

Sanger sequencing of PCR products was performed at a commercial laboratory (Tianyihuiyuan Beijing, China).

### Sequence analysis

According to an overlapping strategy, sequences were assembled using BioEdit ver 7.2.5 software [48]. The generated sequences were aligned and compared with FPV, CPV and MEV sequences from GenBank database, using the MEGA software package version X [49]. These alignments were then subjected for nucleotide and deduced amino acid sequence analyses as implemented in MEGA X. Viral typing of tested sequences was based on the analysis of key VP2 aa residues discriminating the viral type (FPV/CPV) and the CPV variants [26, 50–52].

### Phylogenetic analysis

To elucidate the evolutionary history of FPV and CPV VP2 sequences identified in this study, a phylogenetic tree was constructed with 67 sequences obtained in this study and 45 sequences corresponding to the full-length VP2 gene from CPV, FPV and MEV strains (GenBank No.: FPV: EF988660, AB054226, JX048608, EU498716, EU498720, M24004, MK570646, AF015223, EU252145, EU498681, EU498713, EU252146, EU252147, FJ440712, EU360958; CPV: EU914139, FJ011098, M24003, M74849, FJ005214, FJ005245, FJ005233, HQ025913, KP682522, MH329284, GQ379042, KP090138, MT488457, KT156833, KY418607, MN453226, JX048607, KT162014, MK357727, MK895487, MN453228, KP749854, KT156832, MT488453, MF467242, MK357735, MF467229; MEV: FJ712221, KY094118, KJ186148). In addition, the MEV strain (FJ712221) was used as the outgroup to root the tree. The tree reconstruction was performed with MEGA X using the maximum-likelihood (ML) method according to the Tamura3-parameters model with discrete Gamma distribution (T92 + G), which was selected using the find-best-fit model algorithm in MEGA X. A total of 1000 replicates were used to generate bootstrap values.

### Detection of recombination

To explore the role of genetic recombination in the evolution of FPV and CPV VP2 gene, a dataset of alignments used in the phylogenetic analysis (Sect. 2.4) were examined using various recombination detection methods. The dataset was examined for recombination events using the incorporated recombination detection program 4 (RDP4) package v. 4.101 software, which contains a collection of methods: Bootscan [53], Chimera [54], GeneConv [55], MaxChi [56], RDP [57], SiScan [58], and 3 Seq [59]. The highest acceptable *P* value was set at 0.05. Only sequences that showed a positive recombination event in three or more different methods within the same general region of the alignment were considered potential recombination sequences. Detected recombination events in the RDP4 were then confirmed using the similarity plot and bootscaning analysis in the SimPlot software package v. 3.5.1 [60], with a window and step sizes of 200 bp and 20 bp, respectively. The recombination breakpoints were detected and evaluated by the Kimura-2 parameter (K2P) and the GapStrip models for bootscan analysis similarity plot, respectively. The potential recombination breakpoints were further identified by maximum-likelihood phylogenetic trees construction of different genome segments with MEGA X, as adopted in the Sect. 2.4.

## Data Availability

The data that support the findings of this study are available in GenBank with accession numbers MT270531-MT270597.

## Abbreviations

FPV: Feline Parvovirus
CPV: Canine Parvovirus
PCR: Polymerase Chain Reaction
PBS: Phosphate buffered saline
ML: Maximum-likelihood
RDP4: Recombination detection program 4
nt: Nucleotide
aa: Amino acid
bp: Base Pair
NS: Non-structural protein
